# Explaining the determinants of hookah consumption among women in southern Iran: a qualitative study

**DOI:** 10.1186/s12889-019-7917-4

**Published:** 2019-12-10

**Authors:** Sakineh Dadipoor, Gerjo Kok, Teamur Aghamolaei, Mohtasham Ghaffari, Ali Heyrani, Amin Ghanbarnezhad

**Affiliations:** 1 Cardiovascular Research Center, Hormozgan University of Medical Sciences, Bandar Abbas, Iran; 2 Department of Work and Social Psychology, School of Psychology and Neuroscience, Maastricht University, Maastricht, Netherlands; 3 Environmental and Occupational Hazards Control Research Center, School of Public Health, Shahid Beheshti University of Medical Sciences, Tehran, Iran; 4 Social Determinants in Health Promotion Research Center, Hormozgan Health Institute, Hormozgan University of Medical Sciences, Bandar Abbas, Iran; 5 Social Determinants in Health Promotion Research Center, Hormozgan Health Institute, Hormozgan University of Medical Sciences, Bandar Abbas, Iran

**Keywords:** Hookah, Smoking, Water pipe, Qualitative research, Women

## Abstract

**Background:**

The prevalence of hookah consumption has been on the increase in Iran over the past two decades. This rate is higher among women than men in the south of Iran than other geographical areas. The purpose of this study was to explain the determinants of hookah consumption among indigenous women of Bandar Abbas city, southern Iran.

**Methods:**

This is the first qualitative study with the conventional content analysis approach that has examined the factors affecting the consumption of hookah at all ecological levels in 2018–2019. Participants, with a maximum variation in terms of age, education, occupation, hookah consumption and geographical areas of the city, were selected purposefully to take part in a semi-structured interview. The data were recorded, typed and analyzed according to the framework of the ecological model of health promotion at five levels (intrapersonal, interpersonal, organizational, social and political). The interview continued until data saturation. MAXQDA software version 10 was used for data management.

**Results:**

Interviews were conducted with 56 participants (21 female hookah smokers, 15 female ex- smokers, and 20 experienced experts). A total of eight main categories were extracted from the data including; positive attitude towards hookah consumption, psychosocial needs, sensory charms of hookah, individual factors, family factors, cultural-environmental backgrounds, social-political backgrounds, and economic challenges.

**Conclusions:**

The results indicated the extensive influence of internal and external factors on the consumption of hookah. In order to successfully reduce the consumption of hookah, it is essential to consider intrapersonal, interpersonal, organizational, social and political factors.

## Background

The prevalence of hookah consumption has been on the increase over the past two decades in Iran. This rate is higher in the south of Iran than other geographical areas [1, 2] Hormozgan, the southernmost province of Iran, is near Arab countries such as Kuwait, the United Arab Emirates, and the Kingdom of Saudi Arabia where water pipe smoking is prevalent among the general population [3]. This province ranks third in Iran in terms of the prevalence of hookah consumption [2]. When comparing male and female Hormozgan residents the prevalence of hookah consumption was found to be higher among women than men [4–6].

In an epidemiological investigation in 2017, the prevalence of hookah consumption was reported to be 10.3% among women in Hormozgan which is several times higher than that in other provinces [2]. Moreover, compared to women outside of Iran, the prevalence of hookah consumption was higher among women in the south of Iran, with the rate being 7 to 8% among women in the Eastern Mediterranean region [7], Lebanese women 4% [8], and Pakistani women 4% [9].

According to the latest World Health Organization (WHO) report, tobacco kills more than 8 million people each year. More than 7 million of those deaths are attributed to direct tobacco use while around 1.2 million are non-smokers who are exposed to second-hand smoke [6] The third international conference on water pipe tobacco smoking research: ‘Moving toward Action ‘held in Beirut in 2017, announced that smoking would soon become the biggest cause of preventable death in the world [10] . The Center for Disease Control and Prevention reports that reduced smoking is associated with the rapid increase in other forms of tobacco use, including hookah [11].

Social, psychological and cultural differences, as well as biological and physiological differences between men and women regarding the reason for using various substances have made it more urgent to investigate the reasons for the use of such substances among women [12].

There are studies that have reported more positive attitudes but more dependence on hookah in women than in men [13, 14]. Also, the rate of hookah consumption in women has risen more than men in recent years [5, 15–17] Such an increase could have a major impact on the health of women, the family and the economy, which is why the WHO named the World Tobacco Day 2010; “Gender and Tobacco with Emphasis on Women” [17] . One study showed that the effects of hookah smoking on women are higher than in men [14] Hookah consumption in women is associated with a risk of increased premature menopause, reduced bone density, infertility, ectopic pregnancy, increased infant disease and mortality, intrauterine growth restriction and increased chromosomal disorders [18, 19] Although hookah consumers are of the opinion that hookah consumption is less dangerous than cigarette smoking and can be a safe alternative to cigarettes [20].

In Iran, there are more restrictions on women for smoking cigarettes than hookahs. This is why the use of hookah has become a recreational pastime in many families, especially amongst women [21] The results of a widespread survey in Iran, in 2007, showed that out of the three common forms of tobacco use (hookah, pipe and cigarette) 82.6% of women used hookah, showing hookah consumption as being very common and popular among Iranian women [22].

Although quantitative studies give us useful and important information about the factors affecting the use of hookah, they are not based on the experience and deep understanding of individuals, as factors affecting the use of hookah in each society are closely linked with the cultural context of individuals in that society. However, most of the quantitative studies have focused on factors affecting the use of hookah among students [23] men [24] and general populations [25],but as far as women are concerned, they have mainly identified factors affecting smoking and smoking cessation [26, 27] . Based on available surveys and different databases, a study which has been specifically dedicated to determining the factors affecting the use of hookah among women cannot be found. On the other hand, according to researchers’ knowledge, two qualitative studies in Iran, one qualitative study in the Eastern Mediterranean region and one qualitative study in Canada have examined the factors affecting the use of hookah among women. In the first and second qualitative studies conducted in Iran, determinants of hookah consumption have been limited to individual and family levels, and the external factors affecting hookah smoking have been neglected [12, 28]. In the third qualitative study conducted in the Eastern Mediterranean region, although more factors were identified compared to the two Iranian studies, the environmental factors were not investigated separately [20] In the fourth qualitative study, as with previous studies, quite similar findings were extracted at individual levels [29]. In addition, the above studies have investigated the causes of hookah use only from the perspective of women who were not chronic smokers, while in the present study, the factors affecting hookah consumption have been identified from the perspective of experienced hookah smoking women (at least 7 times per week for 6 consecutive months), as well as female ex- smokers in order to discover wide and diverse views and also to identify environmental factors. Thus, the present study is the first qualitative study conducted in Bandar Abbas-Southern Iran that, in addition to completing the aforementioned studies, has focused on identifying factors affecting the use of hookah at different intrapersonal, interpersonal, organizational, social and political levels.

## Methods

### Study design

A local exploratory study with conventional content analysis approach was conducted to accurately identify the behavioral-environmental factors involved in the consumption of hookah. In the present study, the researchers used individual interviews because, according to several pilot interviews conducted to evaluate the questions, the majority of women preferred to provide information in a private environment. Therefore, focus groups were not used in the present study and data were collected through face-to-face interviews.

### Study sample

In total, 56 people participated in the study, including 21 hookah smokers, 15 ex-hookah smokers and 20 experts in the field of smoking. Entry criteria were [1]; belonging to the indigenous people of Bandar Abbas [2] being an experienced hookah smoking woman who has smoked at least 7 times per week for 6 consecutive months, and/or being an ex-hookah smoker with a previous history of smoking for at least 6 months (7 times a week) before quitting 6 months before the interviews. Also, experts were selected based on entry criteria among those who had rich information on the topic. The ability to communicate and the willingness to share information were other criteria for entering the study. Exclusion criteria included; using other non-hookah tobacco products, not willing to continue the interview at the time of the interview, and having poor information about the topic.

### Data collection

Purposeful sampling with snowball technique was carried out among subjects with a maximum variation in characteristics such as age, occupation, education, and hookah smoking status from different geographical areas of the city.

Firstly, eight regions were identified in different parts of Bandar Abbas (north, south, east and west) which were among the most prevalent hookah consuming areas. Then, the required permissions were obtained from the local council of each area and the first woman with the target experience was identified. Then, the interviewees were approached by the interviewer after the time and place of the interviews were set. The interviews were voice-recorded. The interviews were conducted in a quiet place such as a mosque or house. Each interviewee would introduce the next potential candidate for an interview. The interviews were conducted in each neighborhood in a similar fashion. The sampling from a panel of experts in hookah consumption was purposive and snowball. A visit was paid to the ‘Anti-tobacco consumption organization of Hormozgan’ to identify the first expert in this domain. An appointment was made with the participant at a certain time and place. Then the interviewee was asked to recommend a second interviewee. The sampling was continued until data saturation, where no new data was obtained from the interviews. The duration of interviews was between 45 and 80 min.

### Interview guide

To conduct the interview, a primary version of the interview guide was developed. The guide included two parts; the first part asked for demographic information ،the second part enquired into the obvious and hidden causes of hookah consumption, and women’s beliefs about hookah consumption. After the first five interviews were carried out, participants’ feedback was used to finalize the interview guide. Accordingly, the rest of the interviews were held. Each interview began with the 5 main questions in the interview guide. In the proceeding, follow up questions the subjects were asked to elaborate on the details. Probe questions were asked to explore the depth of the matter. The primary questions of the interview were: ‘How did a hookah find its way into your life? How did you end up a hookah smoker? Why do you smoke a hookah? In your opinion, what would turn someone into a hookah smoker and what would make them continue this habit? Why is the rate of hookah consumption higher among women in Bandar Abbas?’

### Rigor

The researcher tried to strengthen the credibility of the findings by: 1) Allocating sufficient time for data collection (July 2018–May 2019); 2) Presenting the findings to a number of participants to ensure that their comments have been taken into consideration by the researcher (after the feedback no particular change was made in the data); 3) Sending the data to two colleagues (AH, MG) who were experienced in qualitative research, and based on their feedback, categories and sub-categories were reviewed and corrected.

To ensure the validity of the findings, the sub-categories and examples of the codes were sent to two researchers who were not part of the research team and their views contradicted the views of research colleagues. Therefore, an attempt was made to solve this problem by referring to the initial interviews. In order to ensure the transformability of findings, a complete description of the characteristics of participants and the methods and stages of data collection and analysis along with the examples of participations’ statements were prepared.

### Analysis

All interviews were recorded by the first author and then transcribed verbatim. After a detailed and initial analysis of the text of each interview, the next interview was planned. The interviews were reviewed independently by the first author and corresponding author line-by-line with an open coding approach to identify the concepts hidden in the statements of the participants. With the advancement of analysis and continuous comparison of the extracted codes, their similarities and differences were distinguished. Finally, synthesis of findings resulted in categories and sub-categories which were to be merged. The first author and corresponding author reviewed all the extracted codes in the course of a meeting and discussed the categories and subcategories. They were in agreement on the majority of categories and subcategories, and only had different views on a few cases, which, by referring to the initial interviews and re-examining the codes they managed to resolve. The extracted codes were organized by use of MAXQDA software version 10.

### Ethics considerations

This study is part of a PhD thesis in health education and promotion, which has been approved by the Ethics Committee of Bandar Abbas University of Medical Sciences with the code: IR.HUMS.REC.2018.249. Prior to the interviews, the researcher tried to create an appropriate relationship with the participants by introducing herself and her academic degree, and by explaining the purpose of the study. The participants were also assured about the confidentiality of their name and recorded conversations, and the reason they were selected, and consent for recording their voices was obtained from them.

## Result

Out of the 47 women invited to the interview, 36 accepted and 11 women refused to participate in the study, because they did not want their voices to be recorded and also due to their husbands’ opposition. Of the 22 experienced tobacco experts, two experts refused to be interviewed because of their busy work schedule. The remaining subjects enrolled in the study.

The age range of women was between 15 and 67 years old, 40 ± 16.20, and their age at the onset of hookah smoking was between 13 to 38 years old with an average of 20 years old. They all had a history of hookah consumption of between 6 months and 46 years. The frequency of their hookah smoking was reported as being between 1 and 20 times a day with an average of 3 times a day, and 7–200 times a week with an average of 20 times a day. Other demographic information of the participants is provided in Table 1. The work experience of experts ranged from 5 to 28 years, with an average of 20 years work experience.

**Table 1:** Demographic Characteristics of Participants

Variable	Number (Percentage)
Age of the participant	
15–25	9 (25)
26–35	7 (19.4)
36–45	3 (8.3)
46–55	6 (16.7)
55–65	10 (27.8)
66–75	1 (2.8)
Age at the start of smoking	
10–15	8 (22.2)
16–20	10 (27.8)
21–25	10 (27.8)
21–30	4 (11.1)
31–35	3 (8.3)
31–40	1 (2.8)
Marital status	
Single	4 (11.1)
Married	16 (44.4)
divorced	8 (22.2)
Widow	8 (22.2)
Occupation status	
Housewife	25 (69.4)
Employed	10 (27.8)
Retired	1 (2.8)
Education	
Illiterate	8 (22.2)
Elementary school	6 (16.7)
High school	5 (13.9)
Diploma	9 (25)
Academic	8 (22.2)
Residence	
North	11 (30.6)
South	10 (27.8)
West	6 (16.7)
East	4 (11.1)
Center	5 (13.9)

In total, 8 categories and 32 sub-categories emerged from the data analysis in this study, and the volume of data was very large. So, the present authors have published a systematic review of literature on factors involved in hookah consumption among women [30], we decided to ignore the findings that have been reported frequently in previous articles, and focus only on contributing factors that have been discussed less. Thus, “positive attitude” and “sensory charm of hookah” are not discussed, and only 6 categories and 18 sub-categories will be dealt with in this article (Table 2).

**Table 2:** Determinants of hookah consumption among women in Bandar Abbas

Categories	Sub-categories
Perceived needs	Physical and mental dependence
	Gestational aversion
	Climate related desire
Assertiveness	Internal gap
	Self-efficacy
Family factors	Family legacy
	Strict supervision
	Single parenting
Institutional disposition	Tobacco cessation counseling
	Hookah entrepreneurship
Social perception	Perceived restrictions
	Seasonal economy
	Cultural normality
Power relations	Influence of beneficiaries
	Agility of tobacco industry
	Conspiracy beliefs
	Non-participatory policy making
	Agenda setting

### Perceived needs

Women attributed hookah consumption to a series of physical, mental, and environmental- driven needs. As far as they were concerned they believed their perceived internal needs led to the continuation of hookah consumption. This category has 3 sub-categories supported by the following.

#### Physical and mental dependence

According to the ex-consumers, the use of hookah as a recreational pastime, for pleasure and fun, and to calm the nerves is nothing but an excuse, because women become physically and mentally dependent on hookah, and this dependence is justified by external factors.

*"Women who say that they smoke hookah for pleasure, joy or to calm the nerves are making excuses. They do not want to accept they have become addicted and they are physically and mentally dependent on hookah smoking.” (Interviewee No. 24)*

A number of ex-smokers referred to the long periods of smoking and physical habits as reasons for hookah consumption. According to them, long term consumption of hookah has affected their body and brain and it has become a habit for them. In the meantime, a number of young women referred to their short history of hookah consumption as the reason for not being dependent on hookah, and pointed out that since they have not been smoking hookah for a long time, they could easily stop. This somehow reflects the role of physical and mental dependence on hookah.

*"Most women have a long history of hookah use and they are somewhat addicted to smoking. I myself was like that, as I smoked for 20 years. My body and soul was dependent on it." (Interviewee No. 23)*

#### Gestational aversion

Most women referred to gestational aversion as a factor in the initiation of hookah smoking. They said they hated the smell of hookah before pregnancy, but as soon as they got pregnant they found an irresistible tendency towards hookah. According to them, they tended to use hookah with each pregnancy, and this tendency came to an end with the end of their pregnancy and then re-emerged in their second and third pregnancies.

*"When I got pregnant, I had a strong gestational desire towards hookah smoking. I liked its aroma and smell, I even used to smash the coal and eat it. I bought a hookah and started to smoke throughout my pregnancy, I even smoked on the day of my delivery." (Interviewee No. 34)*

#### Climate related desire

Climate was repeatedly referred to by the participants as one of the factors that affect the use of hookahs. Most participants stated that, throughout the year, the weather in their city is very hot and overwhelming, and they do not have suitable conditions for hiking and sports, so they prefer to spend their leisure time in a cool place, like a coffee shop where hookah is served. Also, a number of experts participating in this study acknowledged the impact of weather conditions in each region on the smoking of hookah or any other substances. For example, in the northern cities of the country, muscle aches and pains are very common due to the high humidity, so people take drugs to reduce this pain.

*"The weather in our city is warm in most of the seasons, and we cannot go out when it is warm. Hot weather gives you a suffocating sensation. We get bored at home, so we smoke hookah, or we go out somewhere cool like a coffee shop and smoke hookah there.” (Interviewee No. 10)*

### Assertiveness

A number of participants maintained that to make up for mental, psychological and economic deficiencies, they began to consume hookah, and now they can’t resist smoking. This main category contained two subcategories, which were supported by the following quotes.

#### Internal gaps

The feeling of internal emptiness was another important factor in hookah use. Lack of attention by others, low self-esteem and desolation have led to a sense of emptiness in women, and they have tried to fill these gaps by smoking hookah.

*"At that time, I wanted to fill some of the emptiness inside me. I had some shortcomings; you know my husband was addicted and he did not pay attention to me, he was not looking after himself, and I always had low self-esteem when I was with him. I was embarrassed." (Interviewee No. 20)*

*"Sometimes I felt I was missing someone in my life who I was really interested in, so to fill this gap and stop my husband or someone else from noticing that, I smoked hookah.” (Interviewee No. 16)*

According to the participants, poverty, unemployment and lack of income lead to hookah smoking. The quotes from the participants that confirm this are as follows.

*“When I do not have a desirable economic status and I cannot travel nor have adequate entertainment, I try to make up for them all by smoking hookah.” (Interviewee No. 17)*

#### Self-efficacy

Another reason for hookah use in women was poor self-efficacy. The majority of female consumers referred to being weak-willed and having poor self-efficacy as an important factor for smoking hookah. They stated that they are not able to withstand the temptation of using hookah in difficult circumstances. Also, despite knowing about the complications of hookah, they do not have the ability and will to quit. Most of them pointed out that, if you have a strong will, you can control yourself in tempting situations.

*"The smell of tobacco can be very tempting for me, but if you have a strong will, you can control yourself. If you have a strong will, you will not turn to things like this, but if you have a weak will, you will use anything." (Interviewee No. 3)*

### Family factors

The majority of participants emphasized on the role of the family in beginning to consume hookah in the first place. As they believed, a family pattern, their conditions and upbringing play a key role in orienting them to hookah consumption. Sub-categories will be discussed below.

#### Family legacy

Most participants acknowledged the effect of Family legacy on hookah consumption. According to them, weakness or the weak will of their parents has been transmitted to them, and since they have been born from smoking mothers, they have a tendency towards hookah consumption.

*"The consumption of hookah has been in my blood since childhood, and all my family members are consumers. My dad smokes hookah, cigarettes and even a pipe. So when my father is a consumer, I will be a consumer too because it is in my blood. It’s genetic, like a particular disease." (Interviewee No. 19)*

*“I was a child to parents who were smokers. My father smoked cigarettes and my mother smoked hookahs even when washing the clothes or dishes. I was brought up in such a family. From early childhood, I had a craving for smoking hookah when watching my parents.” (Interviewee No. 20)*

#### Strict supervision

A number of participants referred to strict monitoring and supervision from the family as a factor that influences the use of hookahs. They believed that sometimes severe pressure and monitoring can produce negative results. Thus, they pointed to emotional communication and reasonable monitoring. Also, according to the experts participating in this study, strict supervision and authoritarian controlling parents strips adolescents of creativity and individuality. When a teenager lives in a very strict family, they will not remain creative. There is no sincere relationship in such a family and the relationships are very authoritarian.

*"I have two daughters, and my husband is very strict and hard on them in regard to smoking hookah and many other things. But my daughters go to my sisters every day and smoke hookah there, and they say we do this because our dad does not allow us to. It you forbid your children from doing something, they want that thing more." (Interviewee No. 21)*

#### Single parenting

A number of participants referred to single-parent families as a factor that influences the use of hookah. These women said they felt lonely following the separation from or the death of their mother, and they have looked to their friends who smoke hookahs for emotional support.

"*When my mom died, I became very lonely. When she was alive, she was very cautious. All my mother's family are not very much interested in hookah. My father saw me smoking hookah and he just said don’t smoke too much and was not strict about it. I told him, it is for amusement and he didn’t say any more about it." (Interviewee No. 2)*

### Institutional disposition

Some institutional approaches and practices are also among the determinants of hookah consumption.

#### Cessation counseling

Ineffective tobacco cessation counseling was one of the sub-categories of organizational/institutional influences. The women believed that smoking cessation counseling is not attractive, continuous and effective, and lacks quality to change one’s behavior, because experts are not used in these programs. The experts also referred to such training as an imposed duty, and people are not motivated to participate in them.

*"The training that experts provide to people in reducing tobacco use are not effective, because they are poor and cannot motivate people to quit smoking. Experts should be empowered, as often they cannot answer the consumer’s questions and give a logical response." (Interviewee No. 11)*

*"Experts who are instructors have not been trained scientifically and practically, and do not have enough experience in this regard, because the training they provide does not produce the desired result." (Interviewee No. 23)*

#### Hookah entrepreneurship

Moreover, a number of experts pinpointed that those subjects from an inappropriate socioeconomic level or unsupported women perceive hookah sale as a type of employment. They make a living this way.

*"Households that are not economically well-off sell hookahs with minimum capital. They might have no other choice for employment. Even if they know how to do a certain job, they might have no money to start that with." (Interviewee No. 50)*

### Social perception

According to a number of participants and experts, there are certain unmet social needs that can account for hookah consumption.

#### Perceived restrictions

The research participants thought they had few options for fun and leisure. Thus, hookah consumption can be one choice for having fun in their leisure time.

*"In our society, certain behaviors are perceived negative, especially if done by women. When there is almost no fun, we amuse ourselves with such things as hookahs.” (Interviewee No. 13)*

#### Seasonal economy

Experts believe that, the seasonal nature of the economy was another factor affecting the use of tobacco products, including hookah. Seasonal economy leads to unemployment during some seasons and increases leisure time. For example, people working in the agricultural sector only farm a few months of the year or people in manufacturing sector mainly work in the last three months of the year, so people who work in these sectors are more likely to fill their free time with hookahs.

*"In a society with a seasonal economy, people have more free time and are more likely to entertain themselves with hookah consumption." (Interviewee No. 49)*

#### Cultural normality

The historical-cultural origin of hookah was another factor repeatedly reported by the participants as an effective factor in the use of hookah. According to them, hookahs were ‘born’ in Bandar Abbas; and from ancient times, the people of this area have been involved in making hookahs as potteries and handicrafts. Also, one of the items that every bride must take to her husband’s home is a traditional hookah. Tobacco has been cultivated from ancient times in this city, and the grandparents’ hookahs are handed down to the next generations. People here smoke hookah in times of joy and sorrow, hookahs are a means of entertainment. A participant in this regard stated:

*"If we don’t offer tea to our guests, it’s ok; but if we don’t offer hookahs it is not. We set aside 10 to 20 hookahs in our celebrations, as we offer everyone hookah just like tea." (Interviewee No. 29)*

### Power relations

Power practice by different stakeholders that play a role in the context of hookah consumption, can affect various aspects of the issue.

#### Influence of beneficiaries

Experts with tobacco experience believed that one of the main obstacles to reducing the amount of hookah smoking is the influence of beneficiaries who, because of the profits, have devoted huge amounts of money into encouraging young people to smoke hookahs. They prevent hookahs from being banned.

*"They cannot stop hookahs because it has a large business enterprise. There is a complex and strong backing behind this industry, which is extremely influential. Although there are certain laws made in the congress, beneficiaries would impede the enactment of these laws.” (Interviewee No. 54)*

#### Agility of tobacco industry

The agility of the tobacco industry was another sub-category that many participants emphasized on. The tobacco industry has serious, up-to-date and continuous operations to maintain its profits, and makes every effort to coordinate its activities with the needs and interests of customers. At any time of the day, they meet customer’s needs.

*"The addictive system has good and high motivation because it works for money, so it tries to make more and more benefits every day. Due to the high cost and benefits involved, they work day and night to stay updated with customers’ tastes.” (Interviewee No. 23)*

#### Conspiracy beliefs

There was another issue that the experts and a number of women referred to. They described hookahs as a kind of cultural invasion by foreign countries that are seeking to eliminate vulnerable generations with satellite propaganda. The participants believed that it’s like a Cold War, because most of the women and young people are being targeted.

*"Now, the target of the tobacco industry is young people. Tobacco products are mainly made in China, and the biggest tobacco-producing factory is in China. It’s kind of a Cold War since it mainly targets young people and women." (Interviewee No. 55)*

#### Non-participatory policies

A vast majority of experts maintained that if in decision-making to reduce hookah consumption, where regional conditions are taken into account and a survey of local hookah consumers and local executives is done, we will probably have better executable regulations.

*“In my opinion, we should first and foremost know what it is that the consumer or even non-consumer wants; it would be better if those of the lower ranks be involved in decisions made by high-rank policy-makers. This would yield better results.” (Interviewee No. 38)*

#### Agenda setting

As the majority of participants believed, there are strict inhibitive laws against hookah consumption in Iran. Yet, they are not efficiently executed. Locally speaking, for different reasons, controlling hookah consumption effectively is not much of a concern. Thus, it is often not prioritized. Strict execution of rules and regulations has not been a priority in practice.

*“Unions are granted no authority to provide hookahs for customers. Yet, there are quite a lot of restaurants and coffee-shops that serve hookahs. They actually break the existing laws and are sometimes fined, closed down by force but soon enough they get their businesses again. They are sentenced to a minimum fine which they can very easily pay off from the earnings of hookah services. I think, there is no true determination to stop hookah consumption completely. Laws are not executed as they should be.” (Interviewee No. 41)*

## Discussion

The current qualitative research has examined the factors affecting the use of hookahs in women based on the first step of Intervention Mapping at different socio-ecological levels.

In this study, the majority of ex-consumers acknowledged their psychological dependence on hookahs. In other words, they believed that hookahs are addictive. Very few qualitative studies have pointed to this issue. These results are consistent with the study of Rima Afifi et al. [20]. In regard to this finding, the following points can be considered: First, in the present and the Rima Afifi studies, non-consumer women were included in the study, in addition to consumer women. It seems that, the views of hookah women consumers are different from non-consumers, because in other qualitative studies that only studied the hookah smoking women, the participants believed that hookahs are not addictive and they could quit whenever they wanted [28]. Another hypothesis is that, the present study, contrary to all other qualitative studies, studied ex-women consumers who had both the experience of smoking and quitting hookah smoking. These women are likely to have a more realistic view on this issue compared to current female consumers.

Gestational aversion was reported by a large number of participants as an effective factor in the initiation of hookah smoking. Women had a strong tendency towards the smell of hookahs, ash and water. According to the researchers’ knowledge, this issue has not been reported as an effective factor in the use of hookahs in any of the previous related studies. This finding presumably can be justified by the fact that, pregnant women have likely been exposed to hookah, and this has created an extreme tendency to use hookah. Considering the dominant culture in Bandar Abbas, most of the people around women are hookah smokers. Therefore, it can be argued that in such infected environments, pregnant women were more susceptible to develop gestational aversion to hookah than women who were not exposed to hookah smoke. In a report, eating cigarette ash was one of the cases of gestational aversion during pregnancy [31]. Considering the effects of hookah on a fetus and the pregnant mother, families are advised to keep pregnant women away from hookahs and its smoke. This requires extensive information and ongoing training of women and their families.

The participants referred to the hot weather in Bandar Abbas as another important factor in the expansion of hookah use in that region. It is possible that the effects of weather in such regions, where there are long periods of very hot weather, is stronger. A study in this regard showed that, the water change is significantly related to the mental and physical health of people, as the human body reacts to the fluctuations of weather elements such as temperature, humidity and air pressure, and if the range of fluctuations exceeds a certain limit, a large number of people will likely be physically and psychologically affected [32]. Whereas appropriate weather conditions have a positive effect on happiness. To explain this finding, it may be argued that extreme weather conditions can eliminate the possibility of recreational activities, such as walking, and, when leisure time cannot be filled with such activities, the use of hookahs may look like an attractive substitute. Similarly, some relevant work of research showed that in hot weather, continuous physical work would be exhausting [32].

Another point is that, the rate of clashes, boredom and verbal and physical tensions is likely to be higher in hot weather conditions [33, 34], and this makes people more susceptible to unhealthy behaviors. It is suggested that the authorities should provide healthy recreational activities which are appropriate for the weather of each province.

Another factor that influenced the use of hookah was internal emptiness. In a similar fashion, Sohrabzadeh, Baheiraei et al. conducted a qualitative study and explored the role of psychological and emotional gaps and needs and found them to be involved in why women consumed hookahs [28, 35]. Moreover, Salameh et al. reported the termination of dysphoria as a key reason for women smoking hookahs [14]. It seems that women, who feel an empty gap inside, try to fill it in a variety of ways, such as smoking hookah and creating the sense of satisfaction within themselves. In this regard, a study showed that a significant percentage of participants referred to the sense of hopelessness as an important factor in turning to drug abuse [36].. One of the possible ways to affect this sense of emptiness could be to strengthen spirituality and religious beliefs. In the same vein, Brown and Timberlake showed in a related body of research that attendance in religious meetings once or more a week is associated with less consumption of tobacco [37, 38] Also another study showed that in women, baseline religious activity indirectly reduced tobacco smoking at 24 months by reducing exposure to problem peers at 15 months [39]. It is probable, it seems that strengthening religious beliefs can help to fill the internal gaps among women.

According to the participants, lower self-efficacy was another factor affecting the use of hookah. In some studies, perceived self-efficacy has been identified as one of the most important predictors of non-smoking [40, 41]. In their research, Firoozabadi et al. showed that perceived behavioral control was a predictor of intention for continuing hookah smoking among women. Women of lower perceived behavioral control had the intention of continuing to smoke [40]. This finding can be explained by the fact that women of lower self-efficacy have lower psychological health than others and this could make them react positively to deviations such as hookah consumption. A study found a negative correlation between self-efficacy psychological symptoms ، self-isolation passive and emotional acceptance /avoidance strategies [42]. Also, when dealing with unpleasant and stressful events, people who have high self-efficacy, show more sustainability, and do not accept negative thoughts about themselves and their abilities. Thus, self-efficacy helps them to resist the pressure of friends and control their behaviors [43]. In this regard, a study showed that those who believe they can deal effectively with potential stressors, face and handle stress better, adopt more efficacious coping styles “but if they believe they cannot control aversive events they become distressed and it impairs their level of functioning [44]. It can be useful to develop educational programs to promote self-efficacy using such techniques as verbal encouragement, emotion motivation, modelling and positive skilled experiences as sources for self-efficacy [45] to reduce or stop hookah consumption.

Most participants considered Family legacy to be effective in hookah smoking. From what the participants believed, tendency to smoking hookahs was passed down to them from older generations. As their close relatives were greatly dependent on hookah consumption, this tendency was transferred to them too. A vast majority of participants maintained that upon watching family members smoking, they had one or two puffs too, either overtly or covertly. Then, gradually they tended more and more to smoke hookahs. Participants perceived this tendency as a genetic factor. Yet, the authors see this issue more of a cultural stream within families [7, 27, 46].

A number of participants reported family restrictions as another relevant factor promoting the use of hookah. To explain this finding, it should be noted that in strict families, the emotional relationship between parents and children is probably weak and adolescents who are not intimate with their parents are most affected by such harms. In this regard, a study showed adolescents who rated their parents as having a parenting style with higher levels of intimacy and autonomy, considered a “healthy” parenting style were less likely to initiate smoking, or more likely to report intention to quit if they had already initiated smoking [47]. Thus, it seems effective to educate parents and raise their awareness of how to control children and the unhealthy behavior of hookah consumption.

Single parenting was another issue that was introduced by the participants as a risk factor for hookah use. In the present study, a number of participants referred to the lack of a mother as a risk factor for the use of hookah. Zhang et al. in a study reported that, the smoking rules at home differ between single-parent and two-parent households. In 1995–96, the rate of smoke-free homes was 46% among single-parent households and 63% among the two-parent households. In 2006–07, the rate of smoke-free homes was 75% among single-parent households and 88% among the two-parent households [48].

Concerning the mother’s role in children’s amount of smoking revealed that mother-child relationship, but not father-child relationship, was the significant predictor of smoking status. Also, mother-child relationship could predict low to moderate levels of dependence on nicotine. Finally, among male students, mother-child relationship was the significant predictor of smoking. Neither mother-child nor father-child relationships were the significant predictors of smoking status among female students [49]. It is probable that policy-makers’ attention to the implementation of plans to strengthen family bonds can help to reduce hookah smoking.

Ineffective tobacco cessation counseling was one of the factors at the organizational level mentioned by the participants. It seems that, experienced and trained experts who are not used in programs that tend to reduce tobacco consumption, because they fail to stimulate the motivation of smokers to quit smoking hookah. It is feasible because there has been almost no smoking cessation curriculums for health care providers either in undergraduate or postgraduate programs. In this regard, a study showed that the specialists who received the necessary training were more successful in smoking cessation compared to non-trained specialists [50]. Conducting continuous and effective smoking cessation workshops to empower professionals and motivate them is obvious and necessary.

Hookah entrepreneurship were among the issues that influenced the use of hookahs. In this regard, a study showed that tobacco use in low and middle income countries is higher than in higher income countries [51]. In countries with higher incomes, tobacco control is likely to be better and more effective. In this regard, it can be argued that, low and middle income countries view the supply of hookah as job opportunity, and families who do not have enough capital or do not have a job or any other source of income, will inevitably turn to hookah-related occupations because they can make a living with a small amount of capital. Another hypothesis that can be raised is that people who have economic problems are unlikely to have healthy recreational activities, because healthier recreational activities are more expensive than cheaper ones like hookah. In this regard, some studies pointed to the cost-effectiveness of hookah as a reason for its use [16, 28].

Perceived restrictions was another issue that was introduced by the participants as a risk factor for hookah use. As a possible explanation for this relationship, one can use this hypothesis; people who experience high levels of happiness show less emotional and behavioral problems because after a joyful event, people feel less sadness, depression, tension and anxiety therefore, have less tendency towards the use of hookah. In this regard, a study showed that non- hookah consuming people had a higher happiness score than hookah consuming people [52]. Thus, policies must be made in the country to reduce depression, and institutionalized collective joy. Also, appropriate opportunities must be provided for people to be able to freely express happiness and joy.

Some experts pointed to the seasonal economy in Iran as a reason for the use of hookahs. It is possible that since people are not engaged in occupational activities all throughout the year, they experience more leisure time, so they fill their free time with their favorite activities which could be hookah smoking. If there are no recreational activities, people are more likely to turn to deviant behaviors, such as the use of hookah...In the same vein, a body of research showed that a key reason why university students tended to consume hookahs was as a leisure activity [53, 54]. Probably planning for alternative leisure activities can help them spend their free time more productively and help to reduce hookah consumption.

The Cultural normality of hookahs in Bandar Abbas was another factor frequently mentioned by the participants. Along with this finding, other studies have pointed to the role of culture in the use of hookah [12, 20, 28] Contrary to the present study, in a qualitative study, only a few participants mentioned the role of culture in the use of hookahs [29]. This contradiction can be largely attributed to the geographic location of the two studies, because one of them was carried out in Canada, and the use of hookah in Canada is probably still not defined as a behavioral pattern which has value among people as it is in southern Iran.

In the political sector, the influence of beneficiaries and agility of tobacco industry were among the issues that led to the spread of hookah consumption. In this regard, one study referred to the interventions of the tobacco industry preventing the adoption and enforcement of tight laws by the government as the biggest challenge in reducing tobacco use [55]. Obviously, the tobacco industry, due to its large-scale profit, spends a lot of money on advertising to stop such laws being enforced. The extensive research over the past decades has shown that the advertising activities of the tobacco industry have been an important factor in the tendency of young people towards smoking and preventing them from quitting [56]. Meanwhile unlike the tobacco industry, it seems the prevention system naturally has less power and influence due to its lack of capital.

Another factor influencing the use of hookah was the conspiracy beliefs of foreign countries. One of the tools for these beliefs seems to be the spread of tobacco smoking, especially among women. In this regard, it presumably can be argued that conspiracy beliefs are likely to be a kind of Cold War which has been imposed on Iran.. Also, this massive propaganda is probably targeting women and adolescents in order to trap them in addiction, as a society can be weakened if its young people are weakened. In this regard, a study showed that smoking is the gateway to addiction [57].

From the perspective of experienced experts, non-participatory policies are among the factors influencing the consumption of hookah. Politicians do not seem to consider the opinions of lower-ranking people in reducing the use of hookahs. A key challenge to quitting tobacco consumption is the lacking participation of the target group in smoking cessation programs. Thus, lack of participation is a barrier to tobacco smoking cessation [58]. Considering the opinions and comments of lower-ranking people in the success of smoking cessation programs can probably help to reduce the rate of hookah consumption.

The lack of strict and serious laws to reduce and prevent hookah was another factor found to be effective in expanding supplies and the use of hookahs. In the body of related literature, the lack of strict rules prohibiting hookah consumption was mentioned as a reason for the prevalence of hookah consumption among women [﻿16﻿, 35, 59]. The deterrent and strict laws against hookah seem to have not yet been ratified, or if there is a law, it does not seem to be a deterrent. In this regard, a study showed that the laws that prohibit smoking are more related to cigarettes, and are not sufficiently applied to hookah use [60] .In another qualitative study, participants reported that, there is no deterrence law for supplying hookahs to people under 18 years of age in coffee shops [29]. Increasing taxes, creating more visible warning labels, knowing the effects of hookahs, banning the supply of flavored tobacco, constraining the tobacco production, increasing tobacco prices, and adopting laws on tobacco-free environments in restaurants and coffee shops can be effective in reducing exposure to tobacco smoke [60]. It seems hookah prevention programs need planning and policies to prevent the establishment of such places in the community, probably by imposing restrictions on the provision of such services which would contribute to the rate of hookah smoking being reduced.

### Strengths, limitations, and future research

There were some limitations in this study. Since the interviews were face-to-face, the participants might have provided responses that were socially desirable. Selecting experienced interviewers with high public relation skills and interviewing in a private environment reduced the risk of socially desirable responses to some extent. Like other qualitative researches, researchers’ beliefs may influence the process of study from conceptualization to engagement with participants and interpretation of data [61]. Although in this study, the researcher used an exploratory inductive approach and allowed the categories to be extracted directly from the data, there was a possibility that the interviewees’ views did not cover all the factors affecting the use of hookahs. In order to eliminate this limitation, the interviews continued until data saturation. Moreover, the extracted classes may not be generalized to other parts of the country. Despite these possible limitations, the present study had some strong points, including the fact that the participants were chronic hookah smokers and this enabled them to have rich experience in the subject matter. Women with a previous history of consumption were selected from those who were chronic hookah smokers. In the present study, in addition to the individual factors, the environmental factors were also considered according to the ecological model of health promotion. The vast amount of data and the convergence of most extracted classes with the findings of other studies in Iran and other countries strengthened the findings of this research. It is important for future studies to focus on factors affecting the hookah cessation in order to design effective interventions.

## Conclusions

The results of the present study indicated that hookah consumption in women is a multi-factual phenomenon. Therefore, the design and implementation of hookah preventing interventions based on an ecological model should be multivariate, multifaceted and environmental. Also, in order to successfully reduce the consumption of hookah, it is imperative and inevitable to focus on all intrapersonal, interpersonal, organizational, social and political factors that affect this phenomenon.

## Data Availability

Data is available by the corresponding author on reasonable request.
